# The Effects of Pre-Season and Relationships with Physical, Physiological, Body Composition, and Load Markers: A Case Study Comparing Starters versus Non-Starters from an Elite Female Professional Soccer Team

**DOI:** 10.3390/medicina59122156

**Published:** 2023-12-13

**Authors:** Rafael Oliveira, João Paulo Brito, Renato Fernandes, Ryland Morgans, Susana Alves, Fernando J. Santos, Paula Pinto, Mário C. Espada

**Affiliations:** 1 Sports Science School of Rio Maior–Polytechnic Institute of Santarém, 2040-413 Rio Maior, Portugal; jbrito@esdrm.ipsantarem.pt (J.P.B.); rfernandes@esdrm.ipsantarem.pt (R.F.); salves@esdrm.ipsantarem.pt (S.A.); 2 Life Quality Research Centre (CIEQV), Complexo Andaluz, Apartado, 2040-413 Rio Maior, Portugal; fernando.santos@ese.ips.pt (F.J.S.); paula.pinto@esa.ipsantarem.pt (P.P.); mario.espada@ese.ips.pt (M.C.E.); 3 Research Centre in Sports Sciences, Health and Human Development (CIDESD), 5001-801 Vila Real, Portugal; 4 School of Sport and Health Sciences, Cardiff Metropolitan University, Cardiff CF23 6XD, UK; rmorgans@cardiffmet.ac.uk; 5 Instituto Politécnico de Setúbal, Escola Superior de Educação, 2914-504 Setúbal, Portugal; 6 Faculdade de Motricidade Humana, Universidade de Lisboa, 1499-002 Lisbon, Portugal; 7 Escola Superior Agraria–Instituto Politécnico de Santarém, 2001-904 Santarém, Portugal; 8 Interdisciplinary Centre for the Study of Human Performance (CIPER), Faculdade de Motricidade Humana, Universidade de Lisboa, 1499-002 Lisbon, Portugal

**Keywords:** athletes, geographic information systems, exercise test, athletes, football, load monitoring, jump ability, playing, muscle strength, women

## Abstract

*Background and Objectives*: Research on female soccer players that analyzes playing status is scarce and has previously only examined load monitoring, while other markers, such as physical (i.e., strength, power, and agility), physiological (i.e., maximal oxygen uptake), and body composition (i.e., body fat mass, fat-free mass, body water, and phase angle) markers, warrant further investigation. Thus, the study aims were to (a) compare physical, physiological, body composition, and load markers between starters and non-starters; (b) compare measurements pre- and post-training intervention (five weeks); and (c) analyze any relationships between physical, physiological, body composition, and load markers in an elite female soccer team. *Materials and Methods*: Fourteen first-team players participated in the study (age 23.29 ± 3.19 years, weight 59.14 ± 6.87 kg, height 1.66 ± 0.08 m). Several physical (*n* = 15), physiological (*n* = 1), body composition (*n* = 11), and load markers (*n* = 14) were collected. In addition, participants were sub-divided into starters (*n* = 7) and non-starters (*n* = 7). *Results*: No differences were revealed between starters and non-starters in any of the examined variables. Moreover, following the training intervention, a significantly lower value was found for total body water/fat-free mass ratio (p = 0.043; ES = 0.582). In addition, there were several correlations detected between load and physical/physiological markers (*n* = 28); load and body composition markers (*n* = 6); physical/physiological and body composition markers (*n* = 34); and physical and physiological markers (*n* = 42). *Conclusions:* In conclusion, only a slight tendency of higher load values for starters than non-starters was observed. In addition, no differences in physical, physiological, and body composition markers were found between starters and non-starters, possibly suggesting that five weeks were not enough to improve such variables. Finally, the present results provide novel information assessing the effects of the pre-season in elite female Portuguese soccer players and contribute to a better understanding of the associations between different types of measurements.

## 1. Introduction

Soccer is considered one of the most popular sports worldwide, regarded as the most famous in Europe, and female soccer has been growing in popularity, especially in the last two decades, with professionalism becoming a reality for many teams [1,2,3]. Technical–tactical complexity characterizes soccer as a team sport, and, subsequently, is associated with an increase in the number of competitive moments and training method improvements, intended to optimize preparation for specific players [2]. Women’s soccer has progressed remarkably in recent years, both in terms of the number of players participating and match-play performances. By association, research has subsequently increased recently in women’s soccer [4,5,6], as the knowledge of essential characteristics for successful women’s soccer performance is useful for coaches, physicians, nutritionists, and exercise physiologists to improve the understanding of the female soccer athlete [7].

Coaches play a key role in designing training sessions [8], and thus, training load can be measured internally, as a psychophysiological response, or externally, considering the physical work performed [9]. External load is usually collected by global positioning systems (GPS), global navigation satellite systems, local positioning systems, and inertial measurement units that belong to micro-electro-mechanical systems (which provide a combination of 3D accelerometers, 3D gyroscopes, and 3D magnetometers) [10]. These systems produce performance indicators, such as high-speed running (HSR), player load (PL), accelerations, and decelerations, that allow a comprehensive understanding of the factors that significantly contribute to successful soccer performance [1,2,3].

Competitive soccer matches present numerous intermittent high-intensity and low-intensity moments [1,2,3], therefore significantly stressing players. In women’s soccer, it is important that non-starting players maintain fitness throughout the season in preparation for the high loads per minute that will be required when entering the match, regardless of the stage [11]. Consequently, addressing the relationship between internal and external load markers may provide additional information for coaches and performance staff. Furthermore, a recent systematic review found a positive association with the rating of perceived exertion (RPE), heart rate-derived measures, and external load markers for elite and semi-professional female soccer players and elite/professional and young amateur male soccer players [12].

The Inclusion of both internal and external markers allows a greater understanding of the mechanisms related to training stimulus and recovery, thus providing information on how the training periodization is actually completed [13]. With the addition of other measures, such as body composition (e.g., body fat mass, fat-free mass, body water, and phase angle), more insights can be obtained. In this regard, a recent study by Fernandes et al. [14] suggested that variations in external intensity measures influence body composition variables across the season in professional female soccer players; namely, there were improvements in body fat mass, fat-free mass, intracellular water (ICW), extracellular water (ECW), total body water (TBW), ratios of ECW/TBW, ECW/ICW, and phase angle. However, other research in woman’s soccer highlighted that players generally achieved the highest internal and external intensity on match day 5 (MD-5, five days prior to the next match) and following that, intensity decreased daily until MD [15,16]. Considering the importance of body composition for athletes, frequent assessments should be conducted. This will allow coaches and athletes to understand the impact of body composition on performance throughout the season and adjust training programs accordingly to maximize performance and prevent injuries [7].

To the best of our knowledge, no study to date has focused on the relationships between physical, physiological, body composition, and load markers (in the same research) in a professional female soccer team, examining the effects of five weeks of training (three in the pre-season and two in the in-season) and comparing starters versus non-starters. For instance, when considering playing status, only two studies analyzed internal load measures [17,18]. Fernandes et al. [18] showed no differences between starters and non-starters, while Romero-Moraleda et al. [17] reported higher internal load values for starters than non-starters. Nonetheless, no physical, physiological, body composition, or external load markers were used in either study [17,18].

Therefore, the study aims were to (a) compare physical, physiological, body composition, and load markers between starters and non-starters; (b) compare measurements pre- and post-training intervention (five weeks); and (c) analyze any relationships between physical, physiological, body composition, and load markers in an elite female soccer team. Moreover, the following hypotheses were considered. (a) Starters may present higher values of the examined load markers; (b) five training weeks would improve values of the analyzed markers; and (c) several relationships may exist among the different variables.

## 2. Materials and Methods

### 2.1. Design

This was an observational study design. At the beginning of the pre-season, all players performed physical, physiological, and body composition assessments in accordance with the club’s normal monitoring procedures. Following these initial tests, players were monitored daily for internal and external load during the five-week training period that included four weeks of the pre-season and one week of the in-season. The period of analysis included 28 training sessions, three friendly matches, and two official matches. All physical, physiological, body composition, and load markers were collected at the club’s official training facility.

### 2.2. Participants

Fourteen professional outfield female soccer players from a Portuguese first league club were involved in the study (age 23.2 ± 3.1 years, weight 59.1 ± 6.9 kg, height 1.66 ± 0.08 m). From the 14 players included, six were defenders, three were midfielders, and five were attackers. Four players were national team players.

The inclusion criteria for the study were as follows: (i) participated in at least 80% of training sessions across the five-week period; (ii) completed both study assessments; (iii) did not use dietary supplements during the study, (iv) were un-injured during the course of the study; and (v) did not participate in another training program during the study period. Additionally, the exclusion criteria for the study were as follows: (i) long-term (three months) injury; (ii) player joining the team following the first study assessment; (iii) lack of full, complete training data; and (iv) goalkeepers, due to the large variation in physical demands compared with outfield players. 

In addition, players were considered starters when at least 60 min of match-play in three consecutive matches were completed, while non-starters were categorized as the remaining players [18]. Thus, seven players were considered starters (four defenders, one midfielder, and two attackers) and seven were considered non-starters (two defenders, two midfielders, and four attackers). Only full training data were included for analysis. 

Prior to data collection, participants were fully informed of the study design and signed their informed consent. The study followed the ethical guidelines for human study as suggested by the Declaration of Helsinki. Furthermore, the study was approved by the research Ethics Committee of the Polytechnic Institute of Santarém, Santarém, Portugal (No 24-2022ESDRM, 25 July 2022).

### 2.3. Training Information

Only team pitch-based training and match sessions were included for analysis. All other sessions, individual training sessions, recovery sessions, and rehabilitation training sessions were excluded [19]. 

The planning of all soccer content was cyclical in nature and reflective of modern methods of periodization in elite soccer, and thus, the external physical load experienced by players was undulating across a micro-cycle leading to match-play. The number of days between matches differed [20]. Specifically, there were five micro-cycles (representing five weeks), where the first micro-cycle had 10 training sessions and one friendly match, the second micro-cycle had five training sessions and one friendly match, the third micro-cycle had two training sessions and one friendly match, the fourth micro-cycle had six training sessions and one official match, and the fifth micro-cycle had five training sessions and one official match. 

All training sessions included technical, tactical, physical, and psychological components. All players completed one to two strength- and power gym-based sessions per micro-cycle incorporating upper and lower body and core exercises, although these sessions were not included in the analyses [19]. 

The first and second days post-match (MD+1 and +2) were a day off, and therefore, no GPS data were available. Additional fitness sessions for non-starters were limited to the immediate post-match period and GPS data were collected but not included in the study analysis. The start of the next MD micro-cycle was MD-4, four days prior to competition, and focused on drills designed to develop players’ strength, power, and ability to repeatedly produce explosive actions. This session was devised to improve technical and tactical understanding when ‘out-of-possession’ whilst developing the necessary physical qualities to produce high accelerations and decelerations without decrement. Individual and unit (defense, midfield, attack) practices followed by positional games and small-sided games with goalkeepers in restricted pitch dimensions were delivered. Three days pre-match (MD-3) aimed to tactically prepare players when ‘in-possession’ whilst developing position-specific high-intensity and sprint running capabilities. Practices entailed full-pitch attacking tactical patterns (10v0, 10v4) and large numbered games regularly concluding in 11v11 format (>8v8 plus goalkeepers). The structure of MD-2, two days prior to the match, concentrated on repeating technical–tactical information at low-intensity in various functional pitch areas and dimensions and thus was regarded as an ‘under-loaded’ session considering all key GPS metrics. This session included position-specific passing patterns and then divided players into unit-specific drills for defending or attacking. The final session of the weekly micro-cycle, MD-1, was standardized with no drill variety where the session intended to provide neural stimulation to players whilst also finalizing tactical situations and set-plays. For the reliability and validity of the study, load markers were relativized, meaning that all absolute values obtained in a session were divided by the total training session time. 

### 2.4. Data Collection

All assessments occurred on two separate occasions, interspersed by five weeks. The same evaluators, who have over five years of expertise in this field, conducted the measurements. First, anthropometric and body composition assessments were performed prior to consuming a normal breakfast and conducting the remaining physical assessments. The physical/physiological test sequence was applied. 

The assessments were performed in an ambient temperature and relative humidity of 22–23 °C and 50–60%, respectively. Prior to the physical and physiological assessments, a standardized warm-up (consisting of low-to-moderate running and dynamic stretching of the lower limbs) was performed. For unilateral tests, the dominant leg was determined by assessing which leg was used to strike a ball with the greatest force and accuracy possible [21].

### 2.5. Anthropometric and Body Composition

The anthropometric and body composition measures were obtained with the participants dressed in light clothing without shoes. The participants were further asked to remove all objects that could interfere with the bio-electrical impedance assessment. The participants’ weight and height were measured using a stadiometer with an incorporated scale (Seca 220, Hamburg, Germany) according to standardized procedures [22].

Body composition data were obtained with bio-electrical impedance analysis through Inbody S10 (model JMW140, Biospace Co., Ltd., Seoul, Republic of Korea) according to the manufacturer’s guidelines [23,24] and the recommendations of previous studies [7]. Electrodes were placed on eight tactile points (both thumbs, middle fingers, and ankles) for the multi-segmental frequency analysis. A total of 30 impedance measurements were obtained at frequencies of 1, 5, 50, 250, 500, and 1000 kHz from different segments of the body, including the right and left arms, trunk, and right and left legs. Moreover, three different frequencies (5, 50, and 250 kHz) were used to collect the 15 reactance and PhA measurements from the right and left arms, trunk, and right and left legs, respectively. The variables collected were body fat mass (BFM), soft lean mass (SLM), FFM, intracellular water (ICW), extracellular water (ECW), total body water (TBW), phase angle (PhA, 50 kHz), ECW/TBW ratio, and ECW/ICW ratio.

The measurements were conducted at 8.30 a.m. [7], following a minimum of 8 h of fasting and emptied bladders. The participants adopted a supine position with arms and legs abducted at a 45° angle, and the dorsal surfaces of the right hand and foot were cleaned with alcohol. Participants then rested for 10 min in a quiet room, where eight electrodes were placed on the cleaned surfaces, and measurements were taken. The participants did not exercise or ingest caffeine or alcohol during the 12 h prior to the assessment. Furthermore, participants were only assessed if currently in the luteal phase of the ovulatory menstrual cycle. Otherwise, participants waited until they were in the luteal phase [25].

### 2.6. Physical Assessments

The physical tests included hand grip strength of the dominant hand, jumping ability utilizing the CMJ and DJ, agility using the Illinois agility test, and the 30 m sprint to assess linear speed. The tests were applied in the following sequence: hand grip strength; vertical jump tests; 30 m sprint; agility test (recovery time between 2 and 5 min was provided between each test). All tests were conducted on artificial grass, where participants wore familiar specific soccer shoes. 

#### 2.6.1. Hand Grip Strength 

Maximal isometric strength was determined using a digital hand dynamometer hand grip (Camry 90 kg, Guangdong, China) to assess the strength of the dominant hand. This criterion was implemented as it has previously observed that there is a statistically significant difference between the grip and pinch strengths of dominant and non-dominant hands in favor of the dominant hand [26]. Studies developed in soccer have used the hand grip test as a way to characterize players of different competitive levels [27], to establish a relationship with lower limb strength [28], and as a predictor of injury risk [29]. Three attempts were made with a 1 min rest interval, to ensure that fatigue or learning effects did not influence the test performance. Only the best attempt was used for further analysis. The test was performed in the standing position where players were asked to place arms close to the body and elbows flexed at 90 degrees. The position was maintained during the 5 s period of maximal isometric contraction. The participants were instructed to hit the dynamometer as hard as possible [30].

#### 2.6.2. Jump Tests 

Three attempts were made for each jump. The interval between attempts was 1 min and there was a 3 min interval between the different jumps. For all jump tests, participants started from an upright standing position with hips and knees flexed at approximately 90° with hands remaining fixed on the hips. For the SJ test, following an audible command, participants performed rapid hip and knee extension to execute the jump, without using a countermovement [31,32]. For the CMJ, from the starting position and following an audible command, participants performed rapid hip and knee flexion (approximately 90°), followed by extension of these joints to accomplish the jump [31,32]. For the DJ, participants started from the standardized start position on top of a 30 cm box. Following a sound command, participants stepped from the box, moving vertically downwards to land on the contact mat. On landing, a rapid hip and knee flexion, followed by extension of these joints to perform the final phase of the DJ, was performed [31,32]. Countermovement jump and DJ were repeated unilaterally for dominant and non-dominant legs. To standardize this, the dominant leg was measured first. All jumps were performed on a contact platform (Chronojump, Chronojump Boscosystem, Barcelona, Spain) [33]. The contact platform was attached to specific hardware (Chronopic^®^, Chronojump Boscosystem, Barcelona, Spain). Free software (Chronojump Boscosystem Software version 2.3.0-79, Spain) was used to extract jump height data. The Chronojump system has been previously validated [33].

#### 2.6.3. Eccentric Utilization Ratio (EUR)

The EUR was calculated as the ratio between CMJ and SJ heights and considered an indicator of lower extremity SSC performance in athletes. An ideal EUR of ~1.1 has previously been suggested, in which the CMJ score should be 1.1 × 10% of the SJ [34].

#### 2.6.4. Lower Extremity Stretch-Shortening Cycle (SSC)

The theory underpinning the SSC is that muscles and tendons are able to store elastic energy in the pre-stretch phase of the movement [35]. One example of this is a CMJ, which tendentially produces more force than the SJ. For SSC measurement, the difference between the two types of vertical jumps is used (CMJ-SJ) [35].

#### 2.6.5. Limb Symmetry Index

The symmetry index [36] was calculated utilizing the equation (dominant limb − non-dominant limb)/(dominant limb) × 100.

#### 2.6.6. Sprint Test (30 m)

Following the standardized warm-up routine, sprint performance (30 m) was assessed to evaluate individual maximal speed during a linear sprint. An adhesive tape was marked 30 cm behind the starting line. Players were asked to perform two 30 m sprints from a split stance starting position with the front foot 0.3 m behind the start line and were instructed to complete with maximum effort. The resting interval was set at 2 min between efforts. All tests were conducted in specific soccer shoes familiar to the players. Two experienced assistant coaches used stop watches (Seiko, S056, Tokyo, Japan) to record the sprint time once the participants crossed the 30 m line. The best result was used for data analysis.

#### 2.6.7. Illinois Agility Test

Following the sprint test and the standardized 2 to 5 min recovery period, the Illinois agility test was performed to assess agility on both sides (right side was standardized first). Participants lay prone (head on the start line) with hands placed palm down by the shoulders. On the command ‘Go’, the stopwatch (Seiko, S056, Tokyo, Japan) started, and participants got up as quickly as possible to run forward 10 m around a cone, then diagonally back 10 m. Next, participants ran up and back through a slalom course of four cones. Finally, participants ran another 10 m up and back past the finishing cone, at which time the timer was stopped. The result was recorded by the evaluators with an accuracy of one hundredth of a second and compared to ensure no greater than 0.10 s difference was observed. Two trials were performed with 3 min for recovery between them, and the best time was used for further analysis [37].

### 2.7. Physiological Test

Following the standardized warm-up and jump, sprint, and agility tests, the Yo-Yo Intermittent Recovery Test Level 2 was performed on artificial grass. This was interpreted as the physiological test and was performed last in the test sequence following 10 min of recovery. The test consists of 2 × 20 m shuttle runs, with 10 s of active recovery. Players were positioned on the start line. On the audio CD signal, players run 20 m to the opposite line, turn on the audio beep, and return 20 m to the start line on or slightly before the next beep. Players then receive a 10 s recovery period prior to commencing the next run on the following audio beep. Players repeat this until the increasing running speed can no longer be maintained. Failure to complete the shuttle run on two consecutive occasions resulted in termination of the test for that participant. Cones indicated the start and end of the 20 m lane and the 5 m for active recovery. The final stage and total distance completed for each participant were recorded for analysis. 

The following formula was used to calculate maximal oxygen uptake (VO_2_max) [38]:

VO_2max_ (mL/min/kg) = distance (m) × 0.0136 + 45.3

### 2.8. Internal Load Quantification

The CR-10 Borg’s scale [39] was employed to monitor players’ RPE. Twenty to thirty minutes following each training session, every participant provided a perceived exertion value using a specifically designed Google form by answering the following question: “how intense was the training session?”. The scale varied from 0 to 10 A.U., where each value was rated as 0—nothing at all; 0.5—extremely weak; 1—very weak; 2—weak; 3—moderate; 4—somewhat strong; 5—strong; 7—very strong; and 10—extremely strong. 

The score was used as a subjective measure of internal intensity, RPE. In addition, the duration of the entire training session and/or match in minutes was multiplied by the RPE to generate the session RPE (s-RPE) [40,41]. All participants were already familiarized with the questionnaire from the previous season.

### 2.9. External Load Quantification

A portable 10 Hz GPS device (PlayerTek, Catapult Innovations, Melbourne, Australia) was utilized to produce data relating to training sessions and match-play. This device also incorporated a tri-axial 100 Hz accelerometer. These types of GPS devices seem to be the most valid and reliable in team sports [42]. 

Ten minutes prior to each training session and match, PlayerTek devices were turned on. The devices were placed in a specifically customized vest pocket located on the posterior side of the upper torso fitted tightly to the body, as is typical during training and match-play. The devices were placed and checked by the same staff member on every occasion, and each player wore the same device [43].

The metrics collected for analysis were total distance, HSR (≥15 km/h) [44], numbers of accelerations (acceleration 1, >1–2 m/s; acceleration 2, >2–3 m/s; acceleration 3, >3–4 m/s; acceleration 4, >4 m/s) and decelerations (deceleration 1, <1–2 m/s; deceleration 2, <2–3 m/s; deceleration 3, <3–4 m/s; deceleration 4, <4 m/s) [15], and player load. All variables were analyzed based on the accumulated values for each assessment period. 

### 2.10. Statistical Analysis

Descriptive statistics are presented as mean ± standard deviation (SD). Normality and homogeneity of the different variables were tested using the Shapiro–Wilk and Levene tests, respectively. Only the single leg of CMJ and 30 m sprint (at baseline) and hand grip strength of the non-dominant arm (post-training) did not present normal distribution (p < 0.05). Still, considering the centrality trend of the majority of the variables, an independent *t*-test was used to compare starters versus non-starters. Additionally, a dependent *t*-test was used to compare baseline and post-training assessments. Significant results were considered at p < 0.05. When significant results were detected, Hedges effect size (ES) was performed to determine the effect magnitude through the difference of two means divided by the standard deviation from the data and the following criteria were used: <0.2 = trivial, 0.2 to 0.6 = small effect, 0.6 to 1.2 = moderate effect, 1.2 to 2.0 = large effect, and >2.0 = very large effect [45]. Finally, Pearson’s product–moment correlation coefficient was used between the different types of markers, where the following thresholds were considered: ≤0.1, trivial; >0.1–0.3, small; >0.3–0.5, moderate; >0.5–0.7, large; >0.7–0.9, very large; >0.9–1.0, almost perfect. All statistical procedures were executed in the IBM SPSS Statistics for Windows (version 27.0, IBM Corp, Armonk, NY, USA). 

## 3. Results

Table 1 presents comparisons of external and internal load markers considering player status. No significant differences were observed. 

Table 2 presents comparisons between physical, physiological, and body composition markers considering player status. No significant differences were observed. 

Table 3 shows comparisons between baseline and post-training intervention for physical, physiological, and body composition markers of all players. Only one significant difference was observed for TBW/fat-free mass with an ES = 0.582 (large effect). 

Table 4, Table 5, Table 6 and Table 7 show the relationship between different physical, physiological, body composition, and load markers.

## 4. Discussion

The aims of this study were to (a) compare physical, physiological, body composition, and load markers between starters and non-starters; (b) compare pre- and post-training (following five-week training intervention); and (c) analyze relationships between physical, physiological, body composition, and load markers in elite professional female soccer players from the same team.

In the present study, and when analyzing the study hypotheses, the first hypothesis was not confirmed, as no significant differences were observed. Indeed, only a slight tendency of higher load values for starters than non-starters was observed, although this was not evident across all load markers. In particular, despite evidence that highlighted starters producing higher values in performance indicators such as total distance, HSR, and s-RPE, starters also reported lower values of player load and RPE. Additionally, training duration was similar for both groups (81.68 ± 1.82 min vs. 80.52 ± 1.61 min, starters vs. non-starters, respectively), and in the number of accelerations and decelerations across all four categories of these markers. These findings support the notion that the recent professionalism of women’s soccer may be associated with players starting the pre-season in better physical condition and 60 min in three consecutive matches during the pre-season may not be sufficient to enhance physical condition and differentiate between starters and non-starters. Furthermore, previous research also showed no differences in playing status when using monotony and strain of s-RPE in female soccer players [18] or when relativizing GPS data in male soccer players [46].

When examining the effects of the five-week training intervention and the potential increase in the various analyzed team markers, the study hypothesis was partially confirmed. Specifically, some improvements were observed in the CMJ, SJ, and DJ, and in the single-leg CMJ and DJ, 30 m sprint, agility, and VO_2max_. Nevertheless, hand grip strength did not improve, contrary to body weight and body fat mass alterations. Despite these changes following the five training weeks, none of the results were significantly different. This was also observed when evaluating EUR, SSC, and LSI. These variables may be related to small changes during the pre-season and potentially associated with good physical condition and lower injury risk. Notably, a relevant number of the sample were experienced, international players; thus, this may partly explain the related condition-level results observed and underlines the importance of training monitoring not only for physical condition but also for injury prevention purposes.

When analyzing the different markers considering player status, starter, and non-starter, it is possible to observe that non-starters improved in several markers. For example, for the DJ and single-leg DJ, starters showed decreased performance, while body composition and body fat mass increased in starters and decreased in non-starters. However, the differences were not significant. These results support those found by Espada et al. [47] in high-level senior, professional male soccer players, as the current values for starters and non-starters are associated with EUR, SSC, and LSI, revealing fairly good mean values of lower limb symmetry. Consequently, the present study findings are in agreement with Espada et al. [47], when indicating that screening for muscle strength and asymmetry may be of particular importance for soccer injury prevention, and sports institutions should pay special attention to potential health problems in athletes exposed to high daily training loads.

Our study confirmed the third hypothesis, as several relationships were found between the different variables. Particularly when considering load and physical/physiological markers, between jump assessments and accelerations 4 and decelerations 3. Additionally, focusing on physical and physiological markers, many other significant correlations were found between agility and 30 m sprint, and single CMJ DL, single CMJ NDL, and single DJ DL. These findings highlight previous indications that lower body explosive strength was the main discriminator between players competing at several competitive levels, with elite players performing significantly better than their non-elite peers [27]. Also, another very recent study showed that physical qualities and anthropometry demonstrated greater prediction magnitudes of very high-intensity running (>19 km/h) (65%) and high-intensity running (13–19 km/h) (63%) compared to low-intensity (<13 km/h) (22%) and total (43%) running distances [48]. Moreover, in the present research, VO_2max_ was found to significantly correlate to SJ, CMJ, EUR, SSC, single CMJ DL, and single CMJ NDL. These results emphasize the need for in-depth and accurate analysis of jumping performance during the pre-season, aiming to determine injury risk [47] and evaluate performance enhancement, since important markers for soccer success such as agility, sprint, and VO_2max_ are associated with jump performance [49]. A recent study in women’s soccer reinforces this suggestion, indicating that to increase the total distance covered during match-play, the most important quality to improve is VO_2max_. Furthermore, enhanced VO_2max_ also positively contributes to an increase in high-intensity and very high-intensity running distances, improvements in high-speed (especially knee flexor) maximum force production, body composition, and sprinting speed, which are considered the most important physical variables in national-level women’s official soccer matches [48].

With respect to the relationships between load and body composition markers, HSR was significantly correlated to body composition variables such as body fat mass, ECW/TBW, ECW/ICW, and phase angle. Focusing on physical/physiological and body composition markers, SJ, CMJ, single CMJ DL, single CMJNDL, SSC, and VO_2max_ were all highly correlated with several body composition variables, particularly ECW/ICW, TBW/fat-free mass, and phase angle. Similarly, a relationship was observed between VO_2max_ and soft lean mass, fat-free mass, and intracellular water. Moreover, 30 m sprint performance was significantly correlated with body fat mass. These findings are particularly important for women’s soccer, as it has been previously stated that aerobic fitness can be highly correlated with participation and match-play [50]. Also, Oliveira et al. [7] showed that body composition characteristics improved over the season in women’s soccer. The present study highlights the need for practitioners involved in elite female soccer to consider implementing workload monitoring strategies during training sessions and match-play and to also consider different periods of the soccer season. This has recently been suggested to be significant regarding the monitoring of weekly load of elite female soccer players [17].

Despite the findings of this study, there are some limitations that should be listed. (a) Only one professional soccer team with 14 players was examined, which consequently did not allow the analysis of playing positions due to the small number of participants; (b) we should be cautious in generalizing the results, as the study team belonged to the Portuguese First League, which may differ significantly when compared to other leagues and countries; (c) the short-term study period of five weeks limited the investigation of pre-season effects; and (d) other contextual factors such as a technical/tactical variables would strengthen the study and should be considered in future research. Future studies should also consider analyzing playing positions, different female players’ age categories, and various phases of the competitive season. Furthermore, it would also be interesting to compare physical, technical, and tactical data throughout the season, across different periods of the season, in comparison with training sessions.

## 5. Conclusions

In general, starters presented slightly higher load values compared to non-starters. Additionally, there were no physical, physiological and body composition differences at baseline and following a five-week training intervention between starters and non-starters. Moreover, the hypothesis that five weeks of pre-season training would improve different physical, physiological, body composition, and load markers was not confirmed. Finally, several relationships were confirmed between the variables. Namely, agility, sprint, and VO_2max_ were associated with jumping abilities, which reinforces the importance of jumping performance; HSR was associated with body fat mass, ECW/TBW, ECW/ICW, and phase angle; and several jump tests and VO_2max_ were associated with various body composition variables, which highlights the importance of controlling body composition and hydration levels in female professional soccer players.

## Figures and Tables

**Table 1:** Comparison of load markers between starters and non-starters.

Variables	Starters	Non-Starters	t-Value	p-Value
Training duration (min)	81.68 ± 1.82	80.52 ± 1.61	1.262	0.231
RPE (AU)	5.25 ± 0.46	5.48 ± 0.77	−0.696	0.500
s-RPE (AU)	414.26 ± 46.84	353.31 ± 117.13	1.278	0.225
Total distance (m/min)	49.67 ± 2.36	46.47 ± 9.77	0.841	0.417
HSR (m/min)	4.55 ± 0.95	4.29 ± 0.96	0.521	0.612
Acceleration 1 (nr/min)	1.21 ± 0.15	1.21 ± 0.14	0.043	0.966
Acceleration 2 (nr/min)	0.77 ± 0.06	0.72 ± 0.12	1.023	0.326
Acceleration 3 (nr/min)	0.26 ± 0.01	0.25 ± 0.05	0.301	0.769
Acceleration 4 (nr/min)	0.07 ± 0.02	0.08 ± 0.2	−1.076	0.303
Deceleration 1 (nr/min)	1.08 ± 0.14	1.05 ± 0.18	0.393	0.701
Deceleration 2 (nr/min)	0.69 ± 0.06	0.70 ± 0.10	−0.212	0.835
Deceleration 3 (nr/min)	0.24 ± 0.03	0.25 ± 0.04	−0.530	0.606
Deceleration 4 (nr/min)	0.11 ± 0.01	0.11 ± 0.03	−0.376	0.714
PL (AU/min)	2.42 ± 0.10	2.54 ± 0.29	−1.028	0.324

RPE, rating of perceived exertion; s-RPE, session rating of perceived exertion; HSR, high-speed running; PL, player load; AU, arbitrary units; m/min, meters per minute; nr/min, number per minute.

**Table 2:** Comparison of physical, physiological, and body composition markers between starters and non-starters on both assessment occasions.

Variables	Assessments	Starters	Non-Starters	t-Value	p-Value
Hand grip strength	Baseline	31.99 ± 6.29	30.46 ± 3.01	0.580	0.573
Dominant arm (kg)	Post-training	31.21 ± 5.31	30.21 ± 2.74	0.443	0.666
Hand grip strength	Baseline	30.89 ± 5.79	29.13 ± 3.30	0.698	0.499
Non-dominant arm (kg)	Post-training	28.84 ± 5.50	29.07 ± 4.91	−0.082	0.936
SJ (cm)	Baseline	26.57 ± 3.73	26.96 ± 3.00	−0.213	0.835
	Post-training	27.57 ± 3.29	27.29 ± 3.43	0.159	0.876
CMJ (cm)	Baseline	28.21 ± 4.81	28.43 ± 3.73	−0.093	0.927
	Post-training	28.62 ± 4.04	28.61 ± 4.51	0.06	0.995
EUR (AU)	Baseline	1.06 ± 0.06	1.05 ± 0.04	0.210	0.838
	Post-training	1.03 ± 0.04	1.04 ± 0.05	−0.406	0.692
SSC (%)	Baseline	5.95 ± 5.90	5.29 ± 3.99	0.245	0.810
	Post-training	3.65 ± 4.34	4.58 ± 4.80	−0.377	0.713
DJ (cm)	Baseline	27.31 ± 3.70	29.26 ± 4.23	−0.915	0.378
	Post-training	29.53 ± 3.79	28.26 ± 4.77	0.552	0.591
Single CMJ	Baseline	14.87 ± 3.78	13.89 ± 1.52	0.640	0.534
Dominant leg (cm)	Post-training	14.51 ± 2.18	14.69 ± 2.15	−0.148	0.885
Single CMJ	Baseline	15.40 ± 2.59	14.70 ± 2.50	0.515	0.616
Non dominant leg (cm)	Post-training	15.11 ± 2.28	15.69 ± 2.43	−0.454	0.658
LSI	Baseline	105.48 ± 11.31	105.52 ± 12.18	−0.005	0.996
(CMJ, %)	Post-training	104.38 ± 8.17	106.92 ± 9.15	−0.583	0.570
Single DJ	Baseline	14.31 ± 3.46	13.80 ± 1.92	0.343	0.737
Dominant leg (cm)	Post-training	18.80 ± 1.53	15.56 ± 2.28	−0.728	0.480
Single DJ	Baseline	15.84 ± 3.53	14.53 ± 2.20	0.836	0.419
Non-dominant leg (cm)	Post-training	15.39 ± 1.62	15.84 ± 3.63	−0.304	0.766
LSI	Baseline	111.65 ± 14.27	105.95 ± 16.38	0.694	0.501
(DJ, %)	Post-training	104.08 ± 6.18	101.12 ± 13.39	0.532	0.604
30 m (s)	Baseline	4.92 ± 0.28	4.83 ± 0.23	0.666	0.519
	Post-training	4.89 ± 0.31	4.72 ± 0.24	1.060	0.310
Agility (s)	Baseline	16.34 ± 0.44	16.06 ± 0.27	1.331	0.210
	Post-training	16.20 ± 0.37	16.04 ± 0.33	0.850	0.412
VO_2max_ (mL/kg/min)	Baseline	49.73 ± 1.15	49.41 ± 0.69	0.613	0.551
	Post-training	49.96 ± 1.43	49.72 ± 1.38	0.311	0.761
Body weight (kg)	Baseline	62.50 ± 7.88	55.77 ± 3.69	2.046	0.073
	Post-training	63.80 ± 8.13	55.99 ± 4.29	2.249	0.054
Body fat mass (kg)	Baseline	11.70 ± 2.80	11.91 ± 2.61	−0.148	0.885
	Post-training	12.59 ± 2.55	11.34 ± 3.18	0.807	0.435
Soft lean mass (kg)	Baseline	47.78 ± 7.02	41.24 ± 3.71	2.169	0.058
	Post-training	48.10 ± 6.29	41.97 ± 4.13	2.154	0.052
Fat-free mass (kg)	Baseline	50.80 ± 7.50	43.86 ± 3.92	2.170	0.058
	Post-training	51.21 ± 6.71	44.64 ± 4.37	2.171	0.051
ICW (L)	Baseline	23.34 ± 3.55	20.09 ± 1.75	2.176	0.058
	Post-training	23.51 ± 3.12	20.51 ± 1.99	2.147	0.053
ECW (L)	Baseline	13.70 ± 1.86	11.94 ± 1.19	2.107	0.061
	Post-training	13.79 ± 1.76	12.06 ± 1.23	2.129	0.055
TBW (L)	Baseline	37.04 ± 5.39	32.03 ± 2.96	2.162	0.058
	Post-training	37.30 ± 4.87	32.57 ± 3.20	2.148	0.053
ECW/TBW	Baseline	0.37 ± 0.006	0.37 ± 0.006	−0.771	0.456
	Post-training	0.37 ± 0.004	0.37 ± 0.005	−0.108	0.915
ECW/ICW	Baseline	0.59 ± 0.02	0.59 ± 0.01	−0.708	0.492
	Post-training	0.59 ± 0.01	0.59 ± 0.01	−0.160	0.876
TBW/fat-free mass	Baseline	72.96 ± 0.23	73.03 ± 0.15	−0.740	0.474
	Post-training	72.88 ± 0.12	72.94 ± 0.18	−0.693	0.502
Phase Angle (θ. 50 kHz)	Baseline	6.60 ± 0.58	6.24 ± 0.54	0.752	0.254
	Post-training	6.56 ± 0.37	6.51 ± 0.46	0.565	0.851

SJ, squat jump; CMJ, counter-movement jump; EUR, eccentric utilization ratio; SSC, lower extremity stretch-shortening cycle; DJ, drop jump; LSI, limb symmetry index; VO_2max_, maximal oxygen uptake; ECW, extracellular water; ICW, intracellular water; TBW, total body water; AU, arbitrary units.

**Table 3:** Comparison of physical, physiological, and body composition markers between baseline and post-test.

Variables	Baseline	Post-Test	t-Value	p-Value
Hand grip strength DA (kg)	31.22 ± 4.80	30.71 ± 4.09	0.700	0.496
Hand grip strength NDA (kg)	30.01 ± 4.62	28.96 ± 5.01	1.008	0.332
SJ (cm)	26.76 ± 3.26	27.43 ± 3.23	−1.798	0.095
CMJ (cm)	28.32 ± 4.14	28.62 ± 4.12	−0.653	0.525
EUR (AU)	1.06 ± 0.05	1.04 ± 0.05	0.919	0.375
SSC (%)	5.62 ± 4.86	4.12 ± 4.42	1.054	0.311
DJ (cm)	28.29 ± 3.94	28.89 ± 4.19	−0.720	0.484
Single CMJ DL (cm)	14.38 ± 2.81	14.60 ± 2.09	−0.327	0.749
Single CMJ NDL (cm)	15.05 ± 2.47	15.40 ± 2.28	−0.645	0.530
LSI (CMJ)	105.50 ± 11.30	105.65 ± 7.95	−0.043	0.966
Single DJ DL (cm)	14.06 ± 2.71	15.18 ± 1.91	−0.761	0.102
Single DJ NDL (cm)	15.19 ± 2.91	15.61 ± 2.71	−0.580	0.572
LSI (DJ)	108.80 ± 15.05	102.60 ± 10.14	1.820	0.092
30 m (s)	4.88 ± 0.25	4.81 ± 0.29	0.800	0.439
Agility (s)	16.22 ± 0.38	16.12 ± 0.36	0.852	0.411
VO_2max_ (mL/kg/min)	49.57 ± 0.93	49.85 ± 1.36	−1.240	0.237
Body weight (kg)	59.14 ± 6.87	59.90 ± 7.45	−2.007	0.066
Body fat mass (kg)	11.81 ± 2.60	11.96 ± 2.84	−0.432	0.673
Soft lean mass (kg)	44.50 ± 6.37	45.04 ± 6.02	−1.757	0.102
Fat-free mass (kg)	47.32 ± 6.79	47.92 ± 6.42	−1.834	0.090
Intracellular water (L)	21.71 ± 3.18	22.01 ± 2.96	−1.836	0.089
Extracellular water (L)	12.82 ± 1.75	12.92 ± 1.71	−1.165	0.265
Total Body water (L)	34.54 ± 4.91	34.94 ± 4.66	−1.706	0.112
ECW/TBW	0.37 ± 0.006	0.37 ± 0.005	1.638	0.125
ECW/ICW	0.59 ± 0.02	0.59 ± 0.01	1.442	0.173
TBW/fat-free mass	72.99 ± 0.18	72.91 ± 0.15	2.242	0.043
Phase Angle (θ. 50 kHz)	6.42 ± 0.57	6.54 ± 0.40	−1.055	0.311

SJ, squat jump; CMJ, counter-movement jump; EUR, eccentric utilization ratio; SSC, lower extremity stretch-shortening cycle; DJ, drop jump; LSI, limb symmetry index; VO_2max_, maximal oxygen uptake; ECW, extracellular water; ICW, intracellular water; TBW, total body water; bold denotes significant difference with p < 0.05.

**Table 4:** Correlations between physical/physiological and load markers.

Variables	HG DH	HG NDH	SJ	CMJ	EUR	SSC	DJ	Single CMJ DL	Single CMJ NDL	LSI (CMJ)	Single DJ DL	Single DJ NDL	LSI (DJ)	30 m	Agility	VO_2max_
Training duration	r = 0.228 p = 0.434	r = 0.336 p = 0.240	r = 0.084 p = 0.774	r = 0.016 p = 0.956	r = 0.056 p = 0.849	r = 0.139 p = 0.635	r = −0.088 p = 0.764	r = 0.010 p = 0.972	r = −0.031 p = 0.916	r = −0.082 p = 0.781	r = −0.101 p = 0.731	r = −0.176 p = 0.547	r = −0.153 p = 0.601	r = −0.047 p = 0.874	r = −0.374 p = 0.188	r = 0.319 p = 0.266
RPE	r = 0.026 p = 0.930	r = 0.110 p = 0.930	r = −0.438 p = 0.117	r = −0.440 p = 0.115	r = −0.416 p = 0.139	r = −0.411 p = 0.144	r = −0.199 p = 0.496	r = −0.495 p = 0.072	**r = −0.548** **p = 0.043**	r = −0.166 p = 0.571	r = −0.435 p = 0.120	**r = −0.494** **p = 0.025**	r = −0.368 p = 0.195	r = 0.446 p = 0.110	r = 0.075 p = 0.800	r = −0.349 p = 0.221
s-RPE	r = 0.149 p = 0.610	r = 0.205 p = 0.483	r = −0.433 p = 0.122	r = −0.433 p = 0.122	r = −0.461 p = 0.097	r = −0.486 p = 0.078	r = −0.386 p = 0.173	r = −0.368 p = 0.195	r = −0.478 p = 0.084	r = −0.214 p = 0.463	r = −0.380 p = 0.180	r = 0.448 p = 0.108	r = −1.93 p = 0.509	r = 0.499 p = 0.069	r = 0.188 p = 0.520	r = −0.375 p = 0.186
Total distance	r = 0.416 p = 0.140	r = 0.508 p = 0.064	r = −0.014 p = 0.961	r = −0.011 p = 0.969	r = −0.041 p = 0.889	r = −0.053 p = 0.856	r = −0.127 p = 0.665	r = 0.051 p = 0.862	r = −0.054 p = 0.854	r = −0.245 p = 0.398	r = 0.074 p = 0.801	r = 0.031 p = 0.915	r = −0.067 p = 0.820	r = 0.302 p = 0.295	**r = 0.659** **p = 0.010**	r = 0.202 p = 0.488
HSR	r = 0.202 p = 0.488	r = 0.274 p = 0.344	r = 0.504 p = 0.066	r = 0.496 p = 0.071	r = 0.411 p = 0.144	r = 0.270 p = 0.350	r = −0.044 p = 0.882	**r = 0.664** **p = 0.010**	r = 0.519 p = 0.057	r = −0.297 p = 0.303	**r = 0.551** **p = 0.041**	r = 0.434 p = 0.121	r = −0.044 p = 0.881	r = −0.282 p = 0.328	r = 0.280 p = 0.332	**r = 0.535** **p = 0.049**
Acceleration 1	r = 0.106 p = 0.717	r = 0.195 p = 0.504	r = 0.043 p = 0.884	r = 0.049 p = 0.867	r = 0.081 p = 0.784	r = 0.204 p = 0.483	r = 0.222 p = 0.446	r = −0.280 p = 0.332	r = −0.107 p = 0.717	r = 0.293 p = 0.309	r = −0.219 p = 0.458	r = 0.019 p = 0.950	r = 0.319 p = 0.267	r = 0.366 p = 0.198	r = 0.443 p = 0.198	r = 0.099 p = 0.736
Acceleration 2	r = 0.083 p = 0.777	r = 0.112 p = 0.704	r = 0.253 p = 0.383	r = 0.247 p = 0.395	r = 0.271 p = 0.349	r = 0.367 p = 0.196	r = 0.253 p = 0.382	r = −0.017 p = 0.954	r = 0.201 p = 0.490	r = 0.443 p = 0.113	r = 0.125 p = 0.670	r = 0.299 p = 0.299	r = 0.281 p = 0.331	r = 0.143 p = 0.625	r = 0.403 p = 0.154	r = 0.258 p = 0.374
Acceleration 3	r = 0.246 p = 0.397	r = 0.092 p = 0.753	r = 0.422 p = 0.133	r = 0.439 p = 0.116	r = 0.437 p = 0.118	r = 0.432 p = 0.123	r = 0.317 p = 0.270	r = 0.411 p = 0.144	**r = 0.532** **p = 0.050**	r = 0.255 p = 0.379	r = 0.374 p = 0.187	**r = 0.547** **p = 0.043**	r = 0.329 p = 0.251	r = −0.291 p = 0.312	r = 0.264 p = 0.362	r = 0.530 p = 0.051
Acceleration 4	r = 0.280 p = 0.333	r = 0.147 p = 0.616	**r = 0.650** **p = 0.012**	**r = 0.664** **p = 0.010**	**r = 0.654** **p = 0.011**	**r = 0.554** **p = 0.040**	r = 0.422 p = 0.133	**r = 0.690** **p = 0.006**	**r = 0.673** **p = 0.008**	r = −0.010 p = 0.974	**r = 0.690** **p = 0.006**	**r = 0.650** **p = 0.012**	r = 0.091 p = 0.756	r = −0.443 p = 0.113	r = 0.092 p = 0.754	**r = 0.541** **p = 0.046**
Deceleration 1	r = 0.116 p = 0.694	r = 0.188 p = 0.521	r = 0.062 p = 0.832	r = 0.075 p = 0.799	r = 0.106 p = 0.717	r = 0.212 p = 0.464	r = 0.226 p = 0.436	r = −0.182 p = 0.533	r = −0.101 p = 0.731	r = 0.087 p = 0.768	r = −0.175 p = 0.549	r = −0.019 p = 0.948	r = 0.170 p = 0.560	r = 0.395 p = 0.163	r = 0.474 p = 0.087	r = 0.282 p = 0.329
Deceleration 2	r = −0.026 p = 0.930	r = −0.097 p = 0.741	r = 0.376 p = 0.185	r = 0.397 p = 0.160	r = 0.451 p = 0.106	**r = 0.545** **p = 0.044**	**r = 0.583** **p = 0.029**	r = 0.041 p = 0.889	r = 0.339 p = 0.235	**r = 0.616** **p = 0.019**	r = 0.251 p = 0.388	r = 0.479 p = 0.083	r = 0.422 p = 0.133	r = 0.015 p = 0.960	r = 0.298 p = 0.300	r = 0.148 p = 0.613
Deceleration 3	r = 0.135 p = 0.644	r = 0.211 p = 0.469	**r = 0.693** **p = 0.006**	**r = 0.653** **p = 0.011**	**r = 0.627** **p = 0.016**	**r = 0.557** **p = 0.038**	r = 0.204 p = 0.483	**r = 0.554** **p = 0.040**	**r = 0.641** **p = 0.013**	r = 0.237 p = 0.414	**r = 0.817** **p < 0.001**	**r = 0.656** **p = 0.011**	r = −0.059 p = 0.840	r = −0.391 p = 0.167	r = 0.038 p = 0.897	r = 0.312 p = 0.278
Deceleration 4	r = 0.180 p = 0.538	r = 0.157 p = 0.591	r = 0.311 p = 0.279	r = 0.305 p = 0.289	r = 0.236 p = 0.416	r = 0.117 p = 0.691	r = −0.113 p = 0.702	r = 0.399 p = 0.157	r = 0.414 p = 0.141	r = 0.021 p = 0.944	r = 0.420 p = 0.135	r = 0.339 p = 0.236	r = −0.074 p = 0.802	r = −0.339 p = 0.236	r = 0.130 p = 0.657	r = 0.322 p = 0.262
PL	r = 0.137 p = 0.639	r = 0.180 p = 0.539	r = 0.419 p = 0.136	r = 0.424 p = 0.131	r = 0.380 p = 0.180	r = 0.351 p = 0.218	r = 0.143 p = 0.626	r = 0.347 p = 0.224	r = 0.499 p = 0.069	r = 0.239 p = 0.411	r = 0.400 p = 0.157	r = 0.495 p = 0.072	r = 0.231 p = 0.427	r = −0.384 p = 0.175	r = 0.229 p = 0.430	r = 0.369 p = 0.194

RPE, rating of perceived exertion; s-RPE, session rating of perceived exertion; HSR, high-speed running; PL, player load; HG, hand grip; DH, dominant hand; NDH, non-dominant hand; SJ, squat jump; CMJ, counter-movement jump; EUR, eccentric utilization ratio; SSC, lower extremity stretch-shortening cycle; DJ, drop jump; DL, dominant leg; NDL, non-dominant leg; LSI, limb symmetry index; VO_2max_, maximal oxygen uptake; bold denotes significant correlation with p < 0.05.

**Table 5:** Correlations between body composition and load markers.

Variables	Body Weight	Body Fat Mass	Soft Lean Mass	Fat-Free Mass	ICW	ECW	TBW	ECW/TBW	ECW/ICW	TBW/Fat-Free Mass	Phase Angle
Training duration	r = 0.245 p = 0.398	r = −0.032 p = 0.915	r = 0.284 p = 0.325	r = 0.279 p = 0.335	r = 0.2294 p = 0.304	r = 0.242 p = 0.404	r = 0.276 p = 0.340	r = −0.219 p = 0.451	r = −0.366 p = 0.198	r = −0.010 p = 0.974	r = 0.440 p = 0.116
RPE	r = −0.024 p = 0.935	r = 0.369 p = 0.194	r = −0.167 p = 0.568	r = −0.174 p = 0.552	r = −0.179 p = 0.541	r = −0.140 p = 0.632	r = −0.165 p = 0.572	r = 0.126 p = 0.667	r = 0.244 p = 0.401	**r = 0.655** **p = 0.011**	r = −0.69 p = 0.814
s-RPE	r = 0.257 p = 0.375	r = 0.294 p = 0.308	r = 0.166 p = 0.570	r = 0.160 p = 0.584	r = 0.156 p = 0.593	r = 0.185 p = 0.528	r = 0.167 p = 0.568	r = −0.038 p = 0.897	r = 0.124 p = 0.672	r = 0.494 p = 0.073	r = −0.049 p = 0.868
Total distance	r = 0.438 p = 0.117	r = 0.025 p = 0.933	r = 0.467 p = 0.093	r = 0.464 p = 0.094	r = 0.463 p = 0.095	r = 0.472 p = 0.089	r = 0.468 p = 0.092	r = 0.019 p = 0.948	r = −0.089 p = 0.763	r = 0.071 p = 0.809	r = 0.322 p = 0.262
HSR	r = 0.098 p = 0.738	**r = −0.560** **p = 0.037**	r = 0.333 p = 0.245	r = 0.331 p = 0.248	r = 0.360 p = 0.206	r = 0.255 p = 0.379	r = 0.323 p = 0.260	**r = −0.772** **p = 0.001**	**r = −0.756** **p = 0.002**	r = −0.476 p = 0.085	**r = 0.685** **p = 0.007**
Acceleration 1	r = 0.165 p = 0.572	r = 0.273 p = 0.346	r = 0.066 p = 0.821	r = 0.070 p = 0.813	r = 0.068 p = 0.818	r = 0.066 p = 0.823	r = 0.067 p = 0.819	r = 0.076 p = 0.796	r = −0.054 p = 0.853	r = −0.278 p = 0.336	r = 0.198 p = 0.497
Acceleration 2	r = 0.206 p = 0.481	r = 0.021 p = 0.943	r = 0.212 p = 0.467	r = 0.214 p = 0.462	r = 0.211 p = 0.469	r = 0.213 p = 0.465	r = 0.212 p = 0.466	r = 0.128 p = 0.664	r = −0.068 p = 0.816	r = −0.251 p = 0.387	r = 0.263 p = 0.364
Acceleration 3	r = 0.089 p = 0.763	r = −0.506 p = 0.065	r = 0.299 p = 0.299	r = 0.299 p = 0.299	r = 0.304 p = 0.291	r = 0.283 p = 0.326	r = 0.297 p = 0.302	r = −0.085 p = 0.774	r = −0.213 p = 0.465	r = −0.242 p = 0.404	r = 0.372 p = 0.190
Acceleration 4	r = −0.016 p = 0.956	r = −0.460 p = 0.098	r = 0.169 p = 0.564	r = 0.167 p = 0.569	r = 0.183 p = 0.532	r = 0.135 p = 0.646	r = 0.166 p = 0.571	r = −0.342 p = 0.232	r = −0.349 p = 0.221	r = −0.148 p = 0.614	r = 0.395 p = 0.162
Deceleration 1	r = 0.309 p = 0.283	r = 0.248 p = 0.393	r = 0.232 p = 0.426	r = 0.235 p = 0.419	r = 0.228 p = 0.433	r = 0.239 p = 0.411	r = 0.233 p = 0.424	r = 0.157 p = 0.593	r = −0.014 p = 0.962	r = −0.279 p = 0.335	r = 0.275 p = 0.341
Deceleration 2	r = −0.052 p = 0.859	r = 0.116 p = 0.692	r = −0.108 p = 0.714	r = −0.103 p = 0.725	r = −0.107 p = 0.716	r = −0.100 p = 0.733	r = −0.105 p = 0.721	r = 0.140 p = 0.633	r = 0.038 p = 0.897	r = −0.242 p = 0.405	r = 0.091 p = 0.757
Deceleration 3	r = −0.026 p = 0.929	r = −0.289 p = 0.316	r = 0.089 p = 0.762	r = 0.087 p = 0.767	r = 0.104 p = 0.723	r = 0.052 p = 0.860	r = 0.085 p = 0.771	r = −0.277 p = 0.337	r = −0.386 p = 0.173	r = −0.127 p = 0.665	r = 0.333 p = 0.244
Deceleration 4	r = −0.039 p = 0.895	**r = −0.639** **p = 0.014**	r = 0.219 p = 0.451	r = 0.214 p = 0.462	r = 0.225 p = 0.439	r = 0.195 p = 0.504	r = 0.215 p = 0.460	r = −0.246 p = 0.397	r = −0.288 p = 0.318	r = 0.053 p = 0.858	r = 0.342 p = 0.231
PL	r = −0.196 p = 0.502	r = 0.519 p = 0.057	r = −0.003 p = 0.991	r = −0.004 p = 0.990	r = 0.014 p = 0.963	r = −0.050 p = 0.865	r = −0.009 p = 0.975	r = −0.321 p = 0.264	r = −0.454 p = 0.103	r = −0.373 p = 0.189	r = 0.457 p = 0.101

RPE, rating of perceived exertion; s-RPE, session rating of perceived exertion; HSR, high-speed running; PL, player load; ECW, extracellular water; ICW, intracellular water; TBW, total body water; bold denotes significant correlation with p < 0.05.

**Table 6:** Correlations between physical/physiological and body composition markers.

Variables	Body Weight	Body Fat Mass	Soft Lean Mass	Fat-Free Mass	ICW	ECW	TBW	ECW/TBW	ECW/ICW	TBW/Fat-Free Mass	Phase Angle
Hand grip strength DH	**r = 0.637** **p = 0.014**	r = 0.053 p = 0.857	**r = 0.673** **p = 0.008**	**r = 0.669** **p = 0.009**	**r = 0.677** **p = 0.008**	**r = 0.660** **p = 0.010**	**r = 0.673** **p = 0.008**	r = −0.309 p = 0.283	r = −0.222 p = 0.446	r = 0.103 p = 0.726	r = 0.342 p = 0.232
Hand grip strength NDH	**r = 0.632** **p = 0.015**	r = 0.172 p = 0.557	**r = 0.620** **p = 0.018**	**r = 0.615** **p = 0.019**	**r = 0.628** **p = 0.016**	**r = 0.593** **p = 0.025**	**r = 0.617** **p = 0.019**	r = 0.304 p = 0.291	r = −0.350 p = 0.291	r = −0.002 p = 0.993	r = 0.422 p = 0.133
SJ	r = 0.243 p = 0.402	r = −0.189 p = 0.518	r = 0.337 p = 0.239	r = 0.339 p = 0.235	r = 0.359 p = 0.207	r = 0.275 p = 0.341	r = 0.330 p = 0.250	r = −0.494 p = 0.073	**r = −0.611** **p = 0.020**	**r = −0.648** **p = 0.012**	**r = 0.603** **p = 0.022**
CMJ	r = 0.264 p = 0.362	r = −0.188 p = 0.519	r = 0.358 p = 0.208	r = 0.361 p = 0.205	r = 0.379 p = 0.181	r = 0.302 p = 0.294	r = 0.352 p = 0.217	r = −0.500 p = 0.069	**r = −0.573** **p = 0.032**	**r = −0.640** **p = 0.014**	**r = 0.587** **p = 0.027**
EUR	r = 0.260 p = 0.370	r = −0.130 p = 0.657	r = 0.331 p = 0.248	r = 0.333 p = 0.244	r = 0.350 p = 0.219	r = 0.278 p = 0.336	r = 0.325 p = 0.257	r = −0.458 p = 0.099	**r = −0.533** **p = 0.050**	**r = −0.603** **p = 0.022**	**r = 0.572** **p = 0.033**
SSC	r = 0.264 p = 0.362	r = −0.034 p = 0.908	r = 0.297 p = 0.303	r = 0.300 p = 0.298	r = 0.315 p = 0.272	r = 0.247 p = 0.394	r = 0.292 p = 0.312	r = −0.395 p = 0.162	r = −0.496 p = 0.072	**r = −0.585** **p = 0.028**	**r = 0.552** **p = 0.041**
DJ	r = 0.210 p = 0.470	r = 0.137 p = 0.640	r = 0.170 p = 0.559	r = 0.173 p = 0.554	r = 0.175 p = 0.549	r = 0.164 p = 0.575	r = 0.172 p = 0.557	r = −0.202 p = 0.488	r = −0.097 p = 0.742	r = −0.258 p = 0.374	r = 0.263 p = 0.363
Single CMJ DL	r = 0.257 p = 0.374	r = −0.429 p = 0.126	r = 0.450 p = 0.106	r = 0.451 p = 0.106	r = 0.471 p = 0.089	r = 0.391 p = 0.167	r = 0.443 p = 0.113	r = −0.572 p = 0.032	**r = −0.590** **p = 0.026**	**r = −0.567** **p = 0.034**	**r = 0.555** **p = 0.040**
Single CMJ NDL	r = −0.003 p = 0.993	r = −0.500 p = 0.069	r = 0.196 p = 0.502	r = 0.198 p = 0.498	r = 0.215 p = 0.460	r = 0.145 p = 0.621	r = 0.190 p = 0.515	r = −0.465 p = 0.094	r = −0.490 p = 0.075	**r = −0.534** **p = 0.049**	r = 0.421 p = 0.133
LSI (CMJ)	r = −0.495 p = 0.072	r = −0.154 p = 0.599	r = −0.476 p = 0.085	r = −0.475 p = 0.086	r = −0.481 p = 0.082	r = −0.455 p = 0.102	r = −0.473 p = 0.088	r = −0.240 p = 0.408	r = 0.244 p = 0.408	r = 0.096 p = 0.743	r = −0.338 p = 0.237
Single DJ DL	r = 0.056 p = 0.850	r = −0.244 p = 0.401	r = 0.156 p = 0.593	r = 0.158 p = 0.589	r = 0.175 p = 0.551	r = 0.109 p = 0.711	r = 0.151 p = 0.606	r = −0.352 p = 0.217	r = −0.472 p = 0.089	r = −0.458 p = 0.100	r = 0.394 p = 0.163
Single DJ NDL	r = 0.098 p = 0.740	r = −0.316 p = 0.272	r = 0.230 p = 0.429	r = 0.232 p = 0.424	r = 0.246 p = 0.396	r = 0.190 p = 0.516	r = 0.227 p = 0.436	r = −0.419 p = 0.136	r = −0.411 p = 0.136	r = −0.482 p = 0.081	r = 0.349 p = 0.222
LSI (DJ)	r = 0.074 p = 0.801	r = −0.160 p = 0.585	r = 0.143 p = 0.626	r = 0.144 p = 0.622	r = 0.146 p = 0.618	r = 0.141 p = 0.630	r = 0.145 p = 0.621	r = −0.232 p = 0.425	r = −0.029 p = 0.921	r = −0.148 p = 0.614	r = 0.007 p = 0.981
30 m	r = 0.504 p = 0.066	**r = 0.707** **p = 0.005**	r = 0.261 p = 0.368	r = 0.262 p = 0.365	r = 0.244 p = 0.401	r = 0.308 p = 0.284	r = 0.268 p = 0.355	r = 0.246 p = 0.396	r = 0.340 p = 0.234	r = 0.242 p = 0.405	r = −0.104 p = 0.723
Agility	r = 0.380 p = 0.181	r = 0.201 p = 0.491	r = 0.329 p = 0.251	r = 0.331 p = 0.248	r = 0.322 p = 0.261	r = 0.352 p = 0.218	r = 0.334 p = 0.244	r = −0.057 p = 0.845	r = 0.081 p = 0.783	r = −0.031 p = 0.917	r = 0.125 p = 0.671
VO_2_max	r = 0.426 p = 0.129	r = −0.316 p = 0.272	**r = 0.588** **p = 0.027**	**r = 0.588** **p = 0.027**	**r = 0.607** **p = 0.021**	r = 0.524 p = 0.055	**r = 0.579** **p = 0.030**	r = −0.507 p = 0.064	**r = −0.643** **p = 0.013**	**r = −0.636** **p = 0.014**	**r = 0.786** **p < 0.001**

DH, dominant hand; NDH, non-dominant hand; SJ, squat jump; CMJ, counter-movement jump; EUR, eccentric utilization ratio; SSC, lower extremity stretch-shortening cycle; DJ, drop jump; LSI, limb symmetry index; DL, dominant leg; NDL, non-dominant leg; VO_2max_, maximal oxygen uptake; ECW, extracellular water; ICW, intracellular water; TBW, total body water; bold denotes significant correlation with p < 0.05.

**Table 7:** Correlations between physical and physiological markers.

Variables	HG DH	HG NDH	SJ	CMJ	EUR	SSC	DJ	Single CMJ DL	Single CMJ NDL	LSI (CMJ)	Single DJ DL	Single DJ NDL	LSI (DJ)	30 m	Agility	VO_2max_
HG DH		r = 0.866 p < 0.001	r = 0.193 p = 0.508	r = 0.242 p = 0.404	r = 0.248 p = 0.394	r = 0.268 p = 0.355	r = 0.365 p = 0.361	r = 0.356 p = 0.211	r = 0.227 p = 0.435	r = −0.258 p = 0.374	r = 0.067 p = 0.820	r = 0.318 p = 0.268	r = 0.467 p = 0.092	r = 0.027 p = 0.928	r = 0.159 p = 0.586	r = 0.280 p = 0.332
HG NDH			r = 0.226 p = 0.437	r = 0.229 p = 0.431	r = 0.216 p = 0.458	r = 0.243 p = 0.402	r = 0.133 p = 0.650	r = 0.307 p = 0.286	r = 0.146 p = 0.619	r = −0.359 p = 0.208	r = 0.154 p = 0.600	r = 0.183 p = 0.530	r = 0.136 p = 0.643	r = 0.058 p = 0.845	r = 0.127 p = 0.664	r = 0.258 p = 0.373
SJ				**r = 0.989** **p < 0.001**	**r = 0.980** **p < 0.001**	**r = 0.942** **p < 0.001**	**r = 0.586** **p = 0.028**	**r = 0.831** **p < 0.001**	**r = 0.834** **p < 0.001**	r = 0.024 p = 0.935	**r = 0.861** **p = <0.001**	**r = 0.806** **p < 0.001**	r = 0.111 p = 0.706	r = −0.393 p = 0.165	r = −0.103 p = 0.727	**r = 0.742** **p = 0.002**
CMJ					**r = 0.991** **p < 0.001**	**r = 0.950** **p < 0.001**	**r = 0.670** **p = 0.009**	**r = 0.856** **p < 0.001**	**r = 0.865** **p < 0.001**	r = 0.032 p = 0.913	**r = 0.846** **p < 0.001**	**r = 0.848** **p < 0.001**	r = 0.205 p = 0.482	r = −0.380 p = 0.180	r = −0.046 p = 0.875	**r = 0.733** **p = 0.003**
EUR						**r = 0.978** **p < 0.001**	**r = 0.733** **p = 0.003**	**r = 0.817** **p < 0.001**	**r = 0.842** **p < 0.001**	r = 0.066 p = 0.822	**r = 0.811** **p < 0.001**	**r = 0.829** **p < 0.001**	r = 0.221 p = 0.447	r = −0.359 p = 0.207	r = −0.076 p = 0.796	**r = 0.728** **p = 0.003**
SSC							**r = 0.782** **p < 0.001**	**r = 0.727** **p = 0.003**	**r = 0.796** **p < 0.001**	r = 0.152 p = 0.605	**r = 0.735** **p = 0.003**	**r = 0.798** **p < 0.001**	r = 0.280 p = 0.332	r = −0.327 p = 0.253	r = −0.116 p = 0.694	**r = 0.686** **p = 0.007**
DJ								r = 0.483 p = 0.080	r = 0.602 p = 0.023	r = 0.241 p = 0.406	r = 0.374 p = 0.187	r = 0.659 p = 0.010	r = 0.575 p = 0.032	r = −0.125 p = 0.670	r = 0.060 p = 0.837	r = 0.384 p = 0.175
Single CMJ DL									**r = 0.889** **p < 0.001**	r = −0.211 p = 0.469	**r = 0.834** **p < 0.001**	**r = 0.804** **p < 0.001**	r = 0.170 p = 0.562	**r = −0.564** **p = 0.036**	r = −0.105 p = 0.721	**r = 0.709** **p = 0.005**
Single CMJ NDL										r = 0.251 p = 0.387	**r = 0.837** **p < 0.001**	**r = 0.937** **p < 0.001**	r = 0.388 p = 0.171	**r = −0.695** **p = 0.006**	r = −0.154 p = 0.600	**r = 0.536** **p = 0.048**
LSI (CMJ)											r = 0.038 p = 0.898	r = 0.318 p = 0.268	r = 0.485 p = 0.079	r = −0.258 p = 0.372	r = −0.087 p = 0.768	r = −0.379 p = 0.181
Single DJ DL												**r = 0.810** **p < 0.001**	r = −0.057 p = 0.847	**r = −0.536** **p = 0.048**	r = −0.115 p = 0.697	r = 0.476 p = 0.085
Single DJ NDL													r = 0.534 p = 0.049	r = −0.529 p = 0.052	r = 0.007 p = 0.981	r = 0.423 p = 0.132
LSI (DJ)														r = −0.138 p = 0.637	r = 0.176 p = 0.546	r = −0.023 p = 0.938
30 m															**r = 0.650** **p = 0.012**	r = −0.199 p = 0.495
Agility																r = −0.009 p = 0.974

HG, hand grip; DH, dominant hand; NDH, non-dominant hand; SJ, squat jump; CMJ, counter-movement jump; EUR, eccentric utilization ratio; SSC, lower extremity stretch-shortening cycle; DJ, drop jump; DL, dominant leg; NDL, non-dominant leg; LSI, limb symmetry index; VO_2max_, maximal oxygen uptake; bold denotes significant correlation with p < 0.05.

## Data Availability

The data presented in this study are available on request from the corresponding author.
